# Collectanea

**Published:** 1832-05

**Authors:** 


### COLLECTANEA.

Floriferis ut apes in saltibus omnia libant,
Omnia nos, itidem, depascimur aurea dicta.

### PHYSIOLOGY.

*Remarks on Absorption by the Skin.* (Extract from the fourth edition of ELLIOTSON'S "BLUMENBACH."

ABSORPTION by the skin, unless friction is employed or the cuticle abraded, has been denied. We are told that Dr. CURRIE'S patient, labouring under dysphagia seated in the œsophagus, always found his thirst relieved by bathing, but never acquired the least additional weight; that Dr. GERARD'S diabetic patient weighed no more after cold or warm bathing than previously; that SEGUIN found no mercurial effects from bathing a person in a mercurial solution, provided the cuticle remained entire; while they occurred when the cuticle was abraded.

But the two former cases are no proofs that water was not absorbed, because the persons immersed did not lose in weight, which they would have done if not immersed, owing to the pulmonary and cutaneous excretions: these, therefore, must have been counterbalanced by absorption somewhere, and no shadow of proof can be urged against its occurrence by the skin, as Dr. KELLIE remarks in his excellent paper on the functions of this part. Seguin, besides, found two grains of the mercurial salt disappear in an hour from the solution, when of the temperature of 72½°.

There is every reason to believe the occurrence of cutaneous absorption independently of friction or abrasion of the cuticle. First, the existence of absorbents all over the surface cannot be intended for use merely, when friction is employed on the cuticle abraded. Secondly, we have many facts which prove absorption without these circumstances, either by the skin or lungs, or both; while no reason can be given why they should be attributed solely to the lungs. A boy at Newmarket, who had been greatly reduced before a race, was found to have gained thirty ounces in weight during an hour, in which time he had only half a glass of wine. Dr. HOME, after being fatigued and going to bed supperless, gained two ounces in weight before seven in the morning. In three diabetic patients of Dr. BARDSLEY'S, the amount of the urine exceeded that of the ingesta, and the body even increased in weight; and in one of the instances as much as seventeen pounds. Dr. CURRIE allows, that in his patient "the egesta exceeded the ingesta in a proportion much greater than the waste of his body will explain; and, indeed, such facts occur every day." The same patient's urine, too, after the daily use of the bath, flowed more abundantly, and became less pungent. KEILL says that he one night gained eighteen ounces in his sleep; and LINING, that, after drinking some punch one cool day, the quantity of humid particles attracted by his skin exceeded the quantity perspired in these two hours and a half by eight and a third ounces," and gives two more such instances in the same table. Dr. EDWARDS observed similar facts in guinea-pigs. Thirdly, we have positive evidence of cutaneous absorption without friction or abrasion, in the case of frogs, toads, nay in scaly lizards, which will increase in weight by cutaneous absorption, even if only a part of them is immersed in water; and remarkably so, if previously made to lose much of their moisture by exposure to the air, although they never surpass the point from which the loss of weight began. The increase is much greater in water than the moistest air.

In all the cases which have been mentioned, there is no reason to suppose that exhalation did not continue both on the skin and in the lungs; so that the absorption must have been greater than it at first sight appears. When no increase of weight has taken place on immersion in the warm bath, absorption must have occurred to maintain the weight, notwithstanding the cutaneous and pulmonary losses; and, when some decrease of weight has been observed, we are not justified in concluding that absorption had not taken place, and not lessened the amount of the loss which would have happened. Indeed, there is no doubt that perspiration is considerably increased in the warm bath. I may remark, that while absorption is more active accordingly as more fluid has been lost, it gradually becomes less as it approaches the habitual standard of plenitude in the individual, and that, while transpiration is increased by elevation, the proportion of absorption is increased by depression of temperature.

Dr. MASSY, of America, about 1812, found that, if the body were immersed in a decoction of madder, this substance became discoverable in the urine by the alkalies; and Dr. ROUSSEAU, in conjunction with Dr. J. B. SMITH, made, in consequence, a number of experiments, from which they conclude that rhubarb and madder are so absorbed, and that these only, of all absorbed substances, can be discovered in the urine, and are seen in that fluid only, and are absorbed by no other parts than the spaces between the middle of the thigh and hip, and between the middle of the arm and shoulder.

### PATHOLOGY.

HACKMAN *on Softening of the Spleen, (Ramollissement, or Spleno-malacie.) —Hecker's Litterarische Annalen der Gesammten Heilkunde.*

WE at present know little concerning diseases of the spleen, although such knowledge must be esteemed a point of great practical importance. Affections of the spleen form a considerable proportion of the disorders peculiar to women; but in low and marshy countries, whether towards the tropics or the poles, they are much more common than with us, and are known as the sequelæ of ordinary intermittent and remittent, and also of yellow fever. The regions most remarkable in this respect are the borders of Denmark, East Friesland, the island of Walcheren, La Vendée, several districts of Hungary (especially Bannat,) and many parts of upper and middle Italy, and, among others, the Pontine marshes and the Campagna near Rome: to these may be added Minorca, South Carolina, Bengal, &c. Of the diseases of the spleen common in the above-named countries, Ramollissement must not be overlooked. The generality of works, however, on the pathology of this organ scarcely mention such a state, and even HESSE himself dismisses it very briefly in his excellent treatise on Ramollissement.

Softening of the spleen is sometimes sporadic, but more frequently it is either epidemic or endemic: in the latter cases it is intimately connected with other diseases which are in themselves epidemic or endemic likewise. *Spleno-malacie* is frequent, not only in the human subject, but is common also among animals, especially among the ruminantia. When sporadic, this affection follows sporadic intermittents; when epidemic, it accompanies epidemic fevers in hot and marshy countries. In the last epidemic which ravaged the north of Germany, softening and hypertrophy of the spleen were phenomena so constant, that Dr. DOHRN even ventured to call the disease *splenitis epidemica contagiosa.* Fevers in Sardinia likewise often terminate in softening of the spleen: this lesion, however, is always the effect of a more general disease; at least, the author has never met with an idiopathic case.

According to the opinion of Hachmann, softening of the spleen depends upon over-congestion, or venous inflammation, of which there are two stages, one of irritation or congestion, and one of true ramollissement. The symptoms of the first are, fever with gastric disorder predominating, but of which the type will vary according to the season, the climate, or the constitution of the patient. In hot and marshy countries, or even in more temperate parallels during the summer, the access of fever is preceded by an ordinary shivering, but the intervals are so slight, that it might perhaps be considered a remittent. The rigors, which vary in duration and intensity, are followed by a burning heat, after which a profuse perspiration prevails. At the beginning of the attack, the patient vomits a clear fluid, often mixed with bile; which ejections, continuing during the cold, cease on the access of the hot stage, and recur with the recurrence of each fit. In northern climates, these symptoms diminish as the disease advances; while, in tropical countries, they continue unabated, as the black vomit of the yellow fever will witness. Hæmatemesis only occurs in chronic cases of spleno-malacie. Another essential symptom of this disease is precordial distress marked by similar exacerbations and remissions with the fever, and which probably depend upon the compression of the diaphragm by the enlarged spleen. This symptom is never entirely absent in any case, and sometimes it is developed in so great intensity as to become truly *orthopnœa.*

Of other diagnostic signs of this disease the principal are, great lassitude, pains in the limbs, vertigo, flushed face and eyes, and great thirst, (which, if satisfied, increases the præcordial pain ;) the tongue at first is of a pale red, and covered by a yellowish fur, by degrees it becomes of a bright red, and cracks; but it remains generally moist, and is often studded with aphthous eruptions.

The abdomen is swelled, soft in the umbilical region, but not tender, pain being only felt when the body is bent and pressure made towards the stomach or spleen. In the epigastrium, a very sensible pulsation may be perceived; and as the disease advances, this pulsation extends also to the region of the spleen. Most frequently the fever is ushered in by diarrhœa, (ten or twelve evacuations in twenty-four hours;) the matter passed being dark coloured, greenish, watery, and very fetid. It is during the febrile exacerbations that the stools are most frequent; and this circumstance, joined to the vomitings, gives to the disease an appearance of cholera. The patients are excited, they sleep but little, wander much, and become delirious; the pulse is variable, at first full and soft, afterwards becoming small and very frequent; sometimes, however, it does not vary much, but remains slow, and occasionally intermitting.

The stage of ramollissement seems to be shewn by a great collapse, and the access of typhoid symptoms, and death ensues as in cases of fever, or follows a state of coma resembling apoplexy. This apoplectic state is a phenomenon that frequently occurs in ramollissement of the spleen.

The progress of *spleno-malacie* is, in general, rapid, like that of the fevers of which it is the result: thus, ramollisement may take place in the course of eleven or twelve days. Still the disease may assume a chronic form, as two cases recorded by Bonet and Portal, and one observed by the author, have sufficiently shewn.

There are different degrees of ramollissement: in the first, the spleen is gorged with blood of a black or dirty brown colour, amongst which the reticular tissues cannot be distinguished; the structure is more friable than when in a healthy state, and pressure will discharge a great quantity of dark-coloured blood, so that there will remain nothing but a sac containing black blood, and a soft matter resembling chocolate.

In the more severe forms of ramollissement, the spleen bursts spontaneously, without any previous violence, during the life of the patient, and the matter escapes into the abdominal cavity, when death speedily ensues. The volume of the viscus thus softened is not always much increased, but most frequently the bulk is considerably augmented if the individual has suffered from endemic intermittent or remittent fever.

Of the mode in which ramollissement of the spleen is effected nothing more is known than of ramollissement in general; however, most pathologists agree that such morbid alterations result from sanguineous congestion and inflammation, disorganising the viscus, and ending in gangrene.

Towards the conclusion of his memoir, the author relates eight cases of acute spleno-malacie, extracted from the writings of Heusinger, Grotanelli, Montfalcon, and Bailly, and one chronic case which fell under his own observation.

---

*Contagion.* In the month of September, 1784, a poor woman died in the hospital at Aberdeen, and was buried in a churchyard in the neighbourhood. A company of young surgeons agreed with the grave-digger to set a mark on the grave, as a direction for them to find the body for anatomical purposes; but some person, in order to disappoint the grave-digger's employers, moved the signal to another grave, that of a woman who had been buried three or four months. The party came, and, directed by the mark agreed on, dug up the grave, drew out the coffin and carried it home. But upon opening it, a vapour like fume of brimstone came forth, and suffocated them in an instant. Two women also going past the room fell down dead, and it was said that eleven persons perished from the baneful effluvia."—*Taylor on Premature Intermittent.*

Cotte, in his Treatise on Meteorology, gives a case of this kind, which happened at Montmorency, in 1773. A vapour arose from the coffin of a person recently buried, during the time of a funeral, and out of 120 persons present,

114 took ill of a putrid and venomous fever. A schoolboy at West Linton, some years ago, getting into a new-made grave, set about to open the projecting corner of a coffin, which as soon as he had penetrated, there issued from thence a strong noxious smell, on which he exclaimed he was suffocated; he revived on being taken out of the place, but fell immediately ill of a pestilential fever, of which he died on the seventh day. By this means a similar fever was communicated to some of the people who had attended him during his illness.—*See Dr. Robertson on the Natural History of the Atmosphere.*

---

*Microscopal Researches on the Blood in Disease.* By M. Donné.

All the world is aware of the avidity with which microscopal researches have been carried on, in the investigation of the intimate structure of the organs, tissues, and fluids of the human body. The blood has been chosen, *par excellence*, as the subject of experiment. We confess that we have hitherto derived little advantage, little useful knowledge, little amusement even, from the various microscopists. No two have looked through the same glasses, and consequently no two have seen the same things; their observations are as numerous as themselves. Yet, as chroniclers of passing events, we are bound to record any fresh inquiries of this kind, though our private opinion of their utility and value may be far from favorable. M. Donné has been examining with the miscroscope the composition of the blood in morbid states of the body. We shall state, as briefly as we can, the results at which he has arrived.

In many diseases the form of the globules is altered. In an individual in good health the globules of the blood are perfectly round, transparent in the centre, and of an equal diameter. In a person worn out by long illness, whose organs have undergone perceptible alterations, the globules are less numerous, smaller, deformed, and their general and regular equality is gone. M. Donné asserts the following facts:

1st. In the blood of a woman twenty-six years of age, dead of gangrene of the lung, and the body emitting the odour of putrefaction, the globules were small and remarkably deformed.

2d. In the blood of a woman dead of puerperal peritonitis, the globules were less deformed than in the preceding instance, but they could not be clearly made out. The fluid effused into the abdomen presented a few, very deformed, globules.

3d. In the serum of the blood of a woman, who had suffered for some time from the disease of the brain, and was bled on account of erysipelas, the globules were very small and few; the blood of the clot did not offer a very regular form.

4th. In the blood of a man who was bled for bilious fever with pneumonia, the globules were fine, and tended to become united together.

5th. Serum of a young girl affected with bilious fever: globules well marked, not very transparent.

6th. Blood of a woman dead of dropsy: globules in very small number.

7th. Blood of a woman dead of disease of the liver: globules tolerable, but disposed to be deformed.

8th. Blood of a young man dead of acute peritonitis, treated by mercurial frictions: globules very deformed.

9th. Blood of another young man under similar circumstances: globules not possessing their natural form, and some amongst them very large.

Appended to the foregoing are some statements concerning the existence of animalculæ in animal fluids. We all know of the discovery, or reputed discovery, of animalculæ in the semen by Spallanzani, and many know that a subsequent physiologist has accurately described their motions and habits. M. Donné has ascertained that these animalculæ do not exist in the secretion of the prostate. M. Donné has discovered in perfectly transparent liquid contained in an ovarian cyst, some very small living animalculæ. They appeared composed of a series of rings, and the termination at each extremity was obscure. They swam slowly by bending their bodies alternately to right and left, and they were of so minute a size, as not to be more than one tenth as large as the spermatic animalculæ. We can only say of these observations, that we hope they may be productive of some utility.—*Journal Hebdomadaire,* No. 40, tome vi., and *Med.-Chirurgical Review.*

---

*A Case attended with singular Circumstances, in which the Operation for Imperforate Vagina was successfully performed.* (From Dr. Stedman's *Contributions to Operative Surgery. Ed. Med. and Surg. Journal.)*

I was called on June 6th, 1831, to see a negro girl, sixteen years of age, a slave of M. Huart, of this island. I found her labouring under a strange complication of diseases. The anus was much bruised, and there were extensive excoriations on each side of it, extending to near the situation of the fourchette of the vagina. The labia pudendi were small, and the left somewhat excoriated.

Between the labia there was no fissure whatever, except near the orifice of the urethra, where there was a small round hole about the size of a large pin's head. The vagina was entirely closed, nor was there the slightest vestige of an opening, and only a very indistinct line where such opening should naturally be. It appeared to be closed by a thick and firm substance covered with skin resembling the other parts of the body. The hole by which the urine passed was soon a good deal enlarged by ulceration, which enabled me to introduce the female catheter, an operation found necessary on the second day of my attendance from retention of urine. On this occasion I drew off a large quantity of fluid of an urinous smell, and mixed with a thin reddish fluid that appeared to be the menstrual discharge. I was confirmed in my opinion that the urine was mixed with the menstrual fluid, for, when I depressed the catheter nearly in a perpendicular direction behind the septum which closed the vagina, a large quantity of fluid of a more distinctly red appearance issued.

I introduced the catheter several times to draw off the urine, and on the three or four first occasions it was mixed with a similar red fluid. Afterwards the urine came away unmixed through the catheter. One of the attendants learned to introduce the catheter, and thus saved me the trouble. After a few days, as the irritation and inflammation of the neighbouring parts diminished, catheterism became unnecessary.

For some time before the operation, the hole through which the urine came ceased to be ulcerated, and contracted to about the size of a large pin's head, so that the catheter could not be introduced.

I treated the sores about the anus in the following manner:

The bowels were kept gently open by small and repeated doses of cold-drawn castor oil. Bread and milk poultices were applied for a few days at the beginning, until the sores began to assume a healthy appearance.

The irritation had been kept up, previous to my being called in, by an ointment composed of lard and red precipitate: this, of course, was discontinued. The patient was put under a gentle course of mercury until the gums were slightly affected, when a little of the decoction of sarsaparilla was given. As the appetite was deficient, the sarsaparilla was succeeded by drinks composed of a slightly bitter infusion of gentian root in cold water. These entirely restored the tone of the stomach.

After continuing the poultices for some days, the sores were washed first with a lotion composed of tincture of myrrh and water, pledgets of lint spread with fatty ointment being applied after each ablution. The entire healing of the sores was completed with the chloride of lime-wash, composed of gr. iv. of the chloride of lime to ℥i. of water mixed with a little rosewater. Every thing being now prepared, I proceeded to the operation for opening the vagina on the 20th of July. I was assisted by my friends, Baron Von Bretton, M.D., Dr. Ravn, deputy king's physician, and Mr. Crooke. There were present, besides, M. Huart and Elias Levy, esq.

*Operation.* The patient was placed on a table in the recumbent posture, a servant sitting behind her and supporting her shoulders. She was held fast, in the position used in the operation of lithotomy, by the gentlemen present.

Having previously ascertained, by examination by the finger per anum, that there was no connexion between the vagina and rectum, I introduced, with some difficulty, a small grooved conductor, in a perpendicular direction, through the small hole above mentioned.

With a small sharp scalpel I then made three rapid incisions of what I judged the requisite length, through the septum and upon the director. The incisions completely divided the septum, which was about a quarter of an inch in thickness, and of a membrano-ligamentous structure. The finger being introduced, the interior of the vagina was found natural in its structure. Of this the medical gentlemen present satisfied themselves.

Scarcely half an ounce of blood was lost, and the operation was completed in about half a minute. A piece of lint steeped in olive oil was introduced on the forefinger between the lips of the wound into the vagina; a compress of lint was applied, and the whole kept in its place by a T bandage. The patient struggled a good deal during the operation.

In the evening I found the patient doing well. The female catheter was introduced by way of precaution, in case the consequent inflammation should close up the orifice of the urethra, and it was fixed by a string to the bandage round the waist.

A similar plan was pursued at each dressing, and the patient is now perfectly recovered, having a vagina of a natural appearance.

*Remarks.* The want of the orifice of the vagina was discovered previous to my attendance by the people employed to dress the sores about the anus. These sores were caused by a *coitus nefandus*, with a negro man, who could not afterwards be discovered. He probably had no intention of committing so grave a crime, but finding the entrance deficient in the usual place, had probably in the heat of passion proceeded to the violence which occasioned the excoriations.

As to the origin of the closure of the vagina, I have no doubt that it took place when the girl was an infant, and that it was caused by excoriations and subsequent union between the nymphæ. This being neglected, had in the course of years formed the complete septum which I have described. I am confirmed in this opinion from the circumstance, that I was called sometime ago to see a similar case in a female mulatto two years of age, in which, from excoriations, the nymphæ were beginning to adhere. The mother, being an intelligent woman, called in medical aid in time, and the adhesions, though extending along the whole orifice of the vagina being slight, I was easily enabled to separate them by the introduction of a silver probe.

The parts were afterwards kept from adhering by pledgets of lint dipped in a weak solution of alum in water.

---

### PRACTICAL MEDICINE.

*Powdered Alum, a Cure for the Toothach.* DR. KUHN asserts that alum, finely powdered, not only relieves the toothach, but that it also arrests the progress of caries in the tooth. One or two grains are to be inserted into the cavity of the tooth, and this is to be repeated when the pain returns: in a short time the pain will cease to recur, and the chemical action which constitutes the caries will cease.—*Gazette Méd. de Paris.*

*Treatment of Prurigo Pudendi.* Many of the celebrated German physicians are in the habit of employing, in cases of this very troublesome affection, a lotion composed of two drachms of chlorate of potash to from six to ten ounces of water. We are assured that much more decided and quicker success has resulted from this remedy than from any other previously suggested by practitioners.

---

*Observations on the Use of Tobacco, as a Local Application in Gout and other Cases of Constitutional Irritation.* By JOHN VETCH, M.D., Physician to the Charterhouse.

UNDER other circumstances it had been my intention to give to the public a series of detailed cases, to establish the beneficial effects of tobacco as a local application, and one capable of alleviating in a great degree, and of sometimes altogether arresting, various forms of specific inflammation, more particularly gout and rheumatic inflammation attacking the synovial membrane. Besides the power which this vegetable possesses in allaying the pain and abating the inflammation of gout, it assists the parts most materially in recovering their tone and strength.

The sensible effects of tobacco upon the skin and cuticle are readily perceived by immersing, for a short time, the fingers in an infusion, or in a watery solution of the extract.

The infusion forms a valuable application in all cases of erysipelatous inflammation; and the only precaution to be attended to is, not to apply it to any part contiguous to the stomach, unless the production of nausea be at the same time desirable.

I was led to appreciate the valuable sedative and astringent power of tobacco in the first instance, by the benefit I derived from it in cases of the last-mentioned class, having many years ago instituted an extensive trial of all the known narcotics, with the expectation of deriving additional aid in the treatment of purulent ophthalmia.

The good and the powerful effects which I obtained from the tobacco fully compensated for the inefficiency of all the other local applications I then tried: its effects were notorious to all who saw it employed, and I now, as I ought to have done twenty years sooner, recommend its use to general notice, in cases of acute migratory inflammation, and especially when it attacks the joints, testicle, or sclerotic coat of the eye.

The infusion as directed by the London Pharmacopœia is sufficiently strong, and in many cases it is well to rub the part with eau de cologne after the use of the tobacco.—*Med.-Chir. Transactions.*

---

### SURGERY.

*Clinical Remarks made by* MR. BRODIE, *Monday, March* 19 *and* 26, 1832.

*Tying of Hemorrhoids.* I AM going, Gentlemen, today to tie some piles, an operation which you have not the opportunity of seeing very often in the hospital. It is one, however, which you will be often called upon to perform in private practice, as the modes of living amongst the higher classes predispose them much more to this complaint than the poor. It is a very safe operation. I never lost more than one case in my private practice, of probably more than three hundred cases in which I have performed the operation. The case which proved fatal was one in which I was obliged to perform the operation, as the patient was used to lose large quantities of blood upon going to stool; and in this case, on which I am now about to operate, Mr. Fermandez informs me that the patient loses about a pint of blood every time he goes to the water-closet. The man is very much blanched in countenance from this excessive hemorrhage, and, being naturally of a dark complexion, he has acquired a sallow, miserable appearance, strongly indicative of debility and suffering. He has also some symptoms of affection of the chest; but it is impossible in his present state to judge how far these go. It is rather a dangerous remedy to tie internal piles; but external piles you may always tie with safety. I used at one time to cut external piles, and this I was led to do from having met with a copy of Mr. Cline's lectures, in which he says, "that cutting off piles is the most safe and certain way of ridding the patient of them; and that none but the most timid surgeons ever tie them; and knowing Mr. Cline to have been a most cautious and careful man in practice, I followed his advice, and cut off piles in several instances; but I was soon obliged to desist from this plan of treatment, owing to the excessive and alarming hemorrhage which came on afterwards. Since then I have always tied piles.

*Hysteric (?) Affection of the Knee.* In a case of inflamed bursa under the ligamentum patellæ, which had been reduced by blistering, but in which the patient still complained of much pain, Mr. Brodie remarked, that the affection which remained about the knee partook more of an hysterical character than any thing else. The patient appeared to shrink when the skin over the knee was pinched up between the fingers, or even before the skin was touched at all. She had also all the appearance of an hysterical person, and the menstrual secretion had never been in proper quantity. Mr. Brodie ordered her Inf. Valerianæ (Ph. D.) ℥xj.; Tinct. Valerianæ Ammon. ℨj. ter in die.

On the 26th this patient was evidently much improved from the valerian; the pain in the knee had almost entirely left her; and Mr. Brodie, in speaking of the use of valerian in these cases, remarked, that Sir Henry Halford, who was "a dexterous prescriber," had a most excellent method of ordering this medicine, which was, in making the infusion, to pour boiling decoction of bark on the valerian root, and letting it stand until it get cold.

*Stricture of the Urethra.* It is very singular (remarked Mr. Brodie) that, although only on Friday last I passed a good-sized bougie, and let it remain for some minutes in the urethra, that now (Monday) it is so firm again, that I cannot pass even a small bougie or a small catheter through it. This man's stricture is situated in the anterior part of the canal, and these strictures are very difficult of cure. They are in general permanent strictures, and are composed of a thick gristly induration of the lining membrane of the urethra; those which are situated farther back in the membranous portion of the urethra, are generally spasmodic and permanent too. The best cure for these gristly indurated strictures, is to introduce a catheter, and cut down upon them at once. To a question asked by some of the pupils, Mr. Brodie replied, that Mr. Stafford's method of curing strictures would not do for these; for in dividing the stricture, you might cut into some of the cells of the corpus spongeosum of the penis, and if the urine get infiltrated there, bad symptoms might arise. Caustic (Mr. Brodie said) was only useful in cases of spasmodic stricture, such as occurred in the membranous portions of the urethra.

*Hospital Gangrene.* I believe hospital gangrene to be nothing more than a common gangrenous ulcer. It derives its name of hospital gangrene more from the circumstance of its appearing frequently in hospitals, than anything else. I have never found it differ in its character from other gangrenous sores.

*Abscess in the Axilla.* An abscess will never heal (remarked Mr. Brodie) unless the opening into it is of sufficient magnitude to allow the matter in it to be discharged as fast as it is formed.

*Aneurism.* When the sac of an aneurism sloughs, the aneurism is always cured, but only by the artery being plugged up above the ligature by a long coagulum. When the old operation for aneurism was performed some years ago, the patients never recovered, for Mr. Pott expressly remarks, that in cases of aneurism it was always thought best surgery to amputate the limb. And the only cases in which the old surgeons would perform the operation (remarked

Mr. Brodie,) were those of popliteal aneurism, for which they made an incision down through the skin and muscle, over the artery, without any minute dissection, then ran a needle almost at random through it, and tied it.

*Dividing the Sphincter Ani.* After performing this operation on a patient, Mr. Brodie remarked, that he should not apply any dressing; merely a poultice. He had a case in private practice of this kind where there were a great many sinuses; but he only applied a poultice, and the case went on very well, and quite recovered. In dividing the sphincter ani muscle for the cure of fistula in ano (said Mr. Brodie) you should completely cut the muscle through, and then the contractions of it only serve to draw the cut edges further apart. If, however, you only partially divide the muscle, the contractions will draw the divided surfaces into closer contact.

*Aneurismal Blood.* In a case of aneurism of the aorta, which is diminishing in size anteriorally under the use of the digitalis and small bleedings, two or six ounces at a time, Mr. Brodie asked whether the blood last drawn exhibited any inflammatory appearance; and, on Mr. Fermandez replying that it had, Mr. Brodie said that it was a remarkable fact, but that aneurismal blood, when drawn from the arm, always bore the marks and shewed the traces of inflammation.—*Lancet.*

---

*Cases in Surgery.* By Robert Logan, Esq. Surgeon, Lanark, and Medical Officer to the Establishment at New Lanark.

1. *Luxation of the Shoulder-joint reduced with unusual facility.* T. H., æt. thirty-four. His left shoulder was luxated by the shaft of a cart falling upon it. He shewed himself at the dispensary within a minute after the occurrence of the accident. On examination, the nature of the accident was evident to the eye, for instead of the natural rounding of the shoulder there was a cavity under the acromion process of size sufficient to have received an egg, and the head of the bone was distinctly felt in the axilla. He could make lateral motion forward and backward, but could not raise his arm. I went out for the purpose of giving directions for its reduction, and returned in a quarter of an hour, when, to my surprise, I was informed it was reduced during my absence. Mr. Cassels, my intelligent assistant, in raising the arm a few inches by the hand, felt the bone slip into its position with a sudden jerk, but receiving no assistance from him beyond the slight elevation mentioned above. The patient himself observed the sudden alteration which had taken place, and expressed himself quite relieved. There is in this man a preternatural relaxation of the articulations, from which I was led to imagine that the capsular ligament had not been ruptured, but merely stretched to the utmost. Considerable swelling arose, accompanied by symptomatic fever, which soon passed off.

2. *Tumor expanding the Spinal Cord and Nerves, without impairing the sensation or motion of the lower extremities.* I was requested by a brother practitioner to see a newly-born infant which had a malformation of the back. On examination, it proved to be a spina bifida of the most extensive description. When first observed, the tumor had, I was informed, a very vascular appearance in the centre; on the edge it was raised up, containing a clear watery fluid, enveloped by a very fine pellucid membrane; the vascular appearance in the centre soon after birth became of a dark purple hue, which remained two days. The surface of the tumor then assumed a sloughy appearance with evident loss of vitality; but the borders were still transparent, through which, at the lower part, the nerves could be distinctly seen passing down in separate bundles towards the outside of the sacrum. Spontaneous rupture took place, and death followed in a few hours on the fourth day.

Leave being granted to examine the body, a crucial incision was made through the membranes enveloping the tumor, and the flaps dissected back. The whole posterior part of the vertebral canal, from the second dorsal vertebra downwards, was wanting. The surface being cleaned, the centre presented an oval mass of a striated fibrous texture, and of a dark purple colour. It was a substitute for the spinal cord, imbedded in cellular substance, which seemed to have taken on a high degree of inflammatory action, every part of it being ramified with enlarged capillary vessels, from which a quantity of florid blood was squeezed by the handle of the scalpel.

Nature had already begun a process for securing and strengthening this most essential part by the deposition of a thick layer of coagulable lymph round its edges, not yet organized. The spinal column maintained its integrity as far down as the second dorsal vertebra; it then degenerated into the mass of nervous substance above described. The osseous fossa, within which this collection of nerves reposed, was from an inch to two inches in width, and lined with a strong tunic, which sent a septum firmly attaching it to the bottom of the hollow throughout its whole length. This tunic was pierced with holes corresponding with others in the bony canal at the junction of each vertebra for the exit of vessels, and for the admission of the intercostal and lumbar nerves. Immediately outside of this tunic, on each side of the spine, there was formed a series of ganglions of a perfectly spherical shape, and about half the size of a garden pea, into which two nervous bundles were seen to pass, separated from each other by the space of a line, and lying in the same plane. It is evident that these must have been the nerves of sensation and of volition of C. Bell; but, from the irregularity of their origin, it was impossible to trace them. At the promontory of the sacrum the nervous bundles were united in a more regular manner, which again underwent subdivision, and were distributed into the holes of the sacrum. The aorta and venæ cavæ were in their usual situation; the kidneys and other viscera of the abdomen were in a natural state. I regret that want of time prevented any examination of the head; but it was well formed, presenting no hydrocephalic symptoms, and the child seemed otherwise in a healthy condition. From such an extensive malformation, paralysis of the lower extremities might have been expected; but it passed its urine and fæces naturally, and when slight pressure was exerted on the tumor, it moved its limbs with tolerable activity.

3. *Rupture of the Recto-abdominal Fascia, exposing the abdominal cavity, yet admitting of delivery terminating favorably.* Mrs. W., æt. thirty-four. About the fourth month of pregnancy, she stated that, from a protracted and severe labour in bearing her last child, she had ruptured her abdomen. On examination, I found that the linea alba had been ruptured from the pubes to within two inches of the xiphoid cartilage. As nothing but the thin integuments intervened betwixt the hand and abdominal organs, it could be readily passed within its walls. The aorta could be distinctly traced, and the uterus and other organs recognized. She laboured under great apprehensions regarding the result of her pregnancy. I recommended to her a broad bandage, which covered the whole of the abdomen, and ending in an obtuse point at the pubes, from which a strap passed betwixt the thighs, and secured at the back, thus affording artificial support in room of the muscles, which had fallen considerably to either side. When labour commenced, the limbs of the infant could be easily traced through the chasm during the intervals of uterine contraction. By the adaptation of a broad bandage, tightened as the labour proceeded, she was enabled to bear her child with tolerable ease in less than twelve hours after the commencement of her illness. The expulsion of the secundines followed in a few minutes afterwards; and she has since done well.

4. *Probable Ossification of the Umbilical Cord, causing hemorrhage.* In a case which lately came under my observation, I had occasion to remark a preternatural fragility of the funis, which, as far as I can recollect, has not been noticed by any obstetric writer. It occurred in a woman of a weak leucophlegmatic temperament. The funis was remarkably short and thick. In tying it previous to the separation of the infant, I was under the necessity of using great precaution, as the smallest degree of undue pressure caused a rupture of the vessels, giving origin to hemorrhage from the infant; indeed, I was obliged to shift the ligature more than once; and in pulling at the cord, in order to assist the expulsion of the placenta from the vagina, it gave way several times. On examination, it appeared of a cartilaginous structure. Query, Might not this state of the parts give rise to serious hemorrhage and death of the child, from any undue pressure during labour?

5. *Tumor of the Pharynx in an Infant, causing violent dyspnœa threatening suffocation, successfully removed by ligature.* A child, æt. three weeks, had since birth been affected with a difficulty of respiration, which produced a croaking noise something similar to that in croup. The infant kept its mouth constantly open, with the tongue retracted, and the point elevated towards the palate. Its colour, when the fits of dyspnœa were most violent, was of a purple hue, and it could not suck without great risk of suffocation. Indeed, from its inability to obtain its natural food, it had undergone a considerable degree of emaciation. On 16th August its mother brought the child to me, and said that she had observed it vomit something red, which filled its mouth, but that it had been immediately swallowed. I was inclined to laugh at the story, especially after examining the child's mouth, when nothing extraordinary was to be seen. By way of experiment, however, I tickled the fauces, so as to induce the action of vomiting, when it brought up, to my great surprise, a tumor three inches in length, and one and a half inches in circumference. Whenever my finger was removed from the mouth it was again swallowed, and the croaking noise recommenced, followed by a considerable discharge of frothy mucus. It was with difficulty that a part of the base of the tumor could be seen, even by depressing the tongue, on account of its deep attachment in the fauces. In its natural situation it occupied the centre of the upper part of the pharynx, having the uvula in front. Its base was of a considerable breadth, as far as could be judged. On the following day, having induced the child to vomit up the tumor, the apex was secured and retained by a tenaculum; a single loop of dentists' silk was then passed through a double cannula, and drawn pretty firmly, after which another was passed to complete the knot; the ends were then cut away, and the tumor swallowed. Next day the tumor was again brought up; but, from the maceration in the mouth, and perhaps the inaccuracy of the application of the ligature, it had slipped. Another single noose was therefore passed over the tumor, tightened by the cannula, and fastened to one of its eyes. The cannula was then retained in the mouth till the circulation was impeded, the ligature being from time to time tightened. The tumor was then cut away by the scissors close to the ligature, and the cannula removed. The child breathed quite freely during the whole time the tumor was in the mouth, and was fed with its mother's milk, which it greedily swallowed from a spoon.

I was obliged to adopt the awkward mode of operating by the cannula, as I did not deem it safe to excise so large a tumor in an infant of such a tender age, and where the difficulty of any remedial application must have been insurmountable; because I concluded that the vessels which fed the tumor must have been of such a size as (when assisted by the warmth and moisture of the mouth) to warrant apprehension of danger. Nor do I conceive that torsion could have here been applied with safety, on account of its broad attachment, as it might have brought away a large portion of the mucous membrane lining the fauces, and done irreparable damage. The base of the tumor now presents a small button-like projection, which does not in the least interfere with the function of the epiglottis. The child is now quite well, and has gained flesh considerably.

The preparation of the tumor I presented to the Royal College of Surgeons of Edinburgh.—*Edinburgh Med. and Surg. Journal.*

### NATURAL PHILOSOPHY.

*Electro-Magnetism.* Mr. Faraday, who was one of the first philosophers who established by direct experiments the intimate connexion of the magnetic and electric forces, by being produced through the agency of common and galvanic electricity many magnetic phenomena, has lately, by a laborious and masterly series of experiments, reversed the proof, and shewn that magnets may be made to develope electric phenomena; and he has even pursued his researches so far, and so successfully, as to exhibit a spark similar to the electric spark from magnets alone.

Mr. Marsh, of Woolwich, has lately shewn that a voltaic battery, that may be contained in a vessel of not larger capacity than a pint, will, when the current is sent through a long coil of wire, or helix, into which a piece of soft iron is introduced, form so powerful an artificial magnet that from it upwards of six hundred weight may be suspended. The fact is not new, but the extent of its power is.

---

*Optics.* Baron Drais, a German nobleman and philosopher, has constructed a very ingenious optical instrument, consisting of two plane mirrors placed parallel to each other, and connected by joints keeping their faces two or three inches apart, and by which the angles of incidence and reflection can be varied to suit any object they are designed to assist in the beholding: by which means, the figures depicted on the one may be seen in the other, and thus a short person is enabled to look over a tall one's head. We have not heard that the Baron has given any name as yet to his apparatus. which is very convenient as to size, for it will fold together, and go into a cardcase. For those who frequent the pits of our theatres, it cannot fail to be of infinite advantage during the Bonnetto mania which now prevails.

---

### MISCELLANEOUS.

*Dietetics.* M. D'Arcet has so far improved Papin's bone digester, that it promises to become a very important economical dietetic apparatus. We learn that several hundred thousand rations of soup have, in consequence of its use, been weekly distributed to the poor of Paris, for some months past: soup equally nutritious with that produced from the meat. Indeed, strange as it may sound, experiments prove that the bones of an animal absolutely afford more gelatine than its flesh; and the jelly, we need not observe, is the only nutritious part of either that is soluble in water. The fat which is separated from the bones, and floats upon the soup, is, when skimmed off, made into candles; and the insoluble portions of the bones are found to produce animal charcoal, purer and of a better quality than that made in the usual way.

